# Therapeutic Potential of *Gentianaceae* Family Plants in the Treatment of Diabetes and Its Complications

**DOI:** 10.3390/biomedicines13112822

**Published:** 2025-11-19

**Authors:** Svetlana Dinić, Melita Vidaković, Jelena Arambašić Jovanović, Aleksandra Uskoković, Nevena Grdović, Marija Đorđević, Jovana Rajić, Mirjana Mihailović

**Affiliations:** Department of Molecular Biology, Institute for Biological Research “Siniša Stanković”—National Institute of Republic of Serbia, University of Belgrade, Bulevar Despota Stefana 142, 11060 Belgrade, Serbia; sdinic@ibiss.bg.ac.rs (S.D.); melita@ibiss.bg.ac.rs (M.V.); jelena.arambasic@ibiss.bg.ac.rs (J.A.J.); auskokovic@ibiss.bg.ac.rs (A.U.); nevenag@ibiss.bg.ac.rs (N.G.); marija.sinadinovic@ibiss.bg.ac.rs (M.Đ.); jovana.rajic@ibiss.bg.ac.rs (J.R.)

**Keywords:** *Gentianaceae* family, diabetes, diabetic complications, antidiabetic, antioxidant, antilipidemic

## Abstract

Diabetes, a metabolic disorder characterized by hyperglycemia resulting from insulin insufficiency or impaired insulin sensitivity, is one of the major global health challenges. Persistent hyperglycemia in diabetes affects microcirculation, eyes, kidneys, liver, pancreas, muscle, and adipose tissue, which consequently leads to irreversible health issues such as retinopathy, nephropathy, neuropathy, cardiovascular complications, abnormalities of lipoprotein metabolism, and gastrointestinal dysfunction. Although available therapies are effective to some extent, they remain limited in efficacy and are often associated with side effects, underscoring the urgent need for novel treatment options. Traditionally, plant extracts and natural compounds have been used for centuries to treat diabetes and its complications. Plant extracts from the *Gentianaceae* family have emerged as a particularly promising source of bioactive compounds proven to be useful for the treatment of various diseases, including diabetes. This review provides a comprehensive overview of the most studied plant extracts and isolated compounds from the *Gentianaceae* family, with a focus on their use in diabetes treatment as well as their action in managing hyperglycemia, antioxidant activity, protection of pancreatic beta cells and associated complications. Numerous in vitro and in vivo studies have demonstrated their great potential to regulate blood glucose levels, reduce oxidative stress, alleviate tissue and organ damage—primarily in the liver and kidney—and improve lipid metabolism. To fully achieve this potential, future research should prioritize well-designed clinical trials to verify safety and efficacy in humans, conduct detailed molecular and cellular studies, standardize extraction and characterization methods to ensure reproducibility, and incorporate conservation biology principles into pharmacognostic investigations.

## 1. Introduction

**Figure 1:**
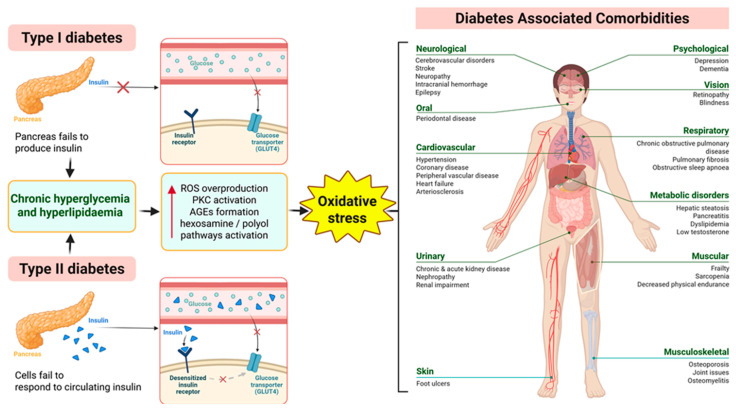
Schematic representation of the relationship between type 1 (T1D) and type 2 (T2D) diabetes, hyperglycemia oxidative stress and diabetic complications. In T1D, autoimmune destruction of pancreatic beta cells leads to insufficient insulin production, while in T2D, peripheral insulin resistance and beta cell dysfunction result in impaired glucose uptake and chronic hyperglycemia and hyperlipidemia. Persistent hyperglycemia and altered carbohydrate and lipid metabolism induce overproduction of reactive oxygen species (ROS) through mitochondrial dysfunction, increased activation of protein kinase C, advanced glycation end products (AGEs), hexosamine and polyol pathway activation and protein kinase C (PKC) signaling. The imbalance between ROS generation and antioxidant defenses leads to oxidative stress, which damages numerous tissues and organs collectively contributing to the progression of diabetic complications including neurological, oral, cardiovascular, urinary, skin, respiratory, eye, metabolic and musculoskeletal disorders.

Diabetes is a metabolic disorder and one of the world’s biggest health problems, reaching epidemic proportions. Diabetes is characterized by hyperglycemia caused by insulin insufficiency due to damage to pancreatic beta cells in type 1 diabetes (T1D) or impaired insulin sensitivity and resistance in type 2 diabetes (T2D), or a combination of both mechanisms over time. According to a World Health Organization report, diabetes impairs quality of life, ability to live and can result in premature death, and it is the seventh leading cause of death [1,2,3,4,5]. Prolonged exposure to elevated plasma glucose levels leads to toxic effects. The most important consequence of hyperglycemia is oxidative stress, i.e., an imbalance between the production and elimination of reactive oxygen and nitrogen species. Accordingly, increased glucose flux leads to increased activation of protein kinase C, an increase in advanced glycation end products (AGEs), activation of hexosamine and polyol pathway, resulting in cell and tissue damage. Chronic hyperglycemia, which is also associated with changes in the genome, epigenome and metabolite markers, leads to long-lasting damage to different levels of the organism. Persistent hyperglycemia in diabetes consequently affects pancreatic beta cells, muscle, liver and adipose tissue (Figure 1). The pathogenesis of diabetes and the adverse effects primarily include retinopathy, nephropathy, neuropathy, cardiovascular complications, abnormalities of lipoprotein metabolism and gastrointestinal dysfunction (Figure 1).

Chronic therapy to maintain normal blood glucose levels is associated with adverse effects [6]. Although conventional therapy has beneficial effects, such as improving blood pressure, lipid profiles and reducing the risk of heart attack, all drugs used in diabetic therapy (insulin sensitizers, sulfonylureas, meglitinides, α-glucosidase inhibitors, glucagon-like peptides 1, amylin analogs) can cause nausea, vomiting and loss of appetite, renal or hepatic insufficiency, an increased risk of cardiovascular death, inflammatory bowel disease, colon ulcers or constipation after short or prolonged use [6]. 

In light of this, the search for new therapeutics is essential, and natural products are an invaluable source. This link to plants underlines the long tradition of using herbal remedies in medicine and remains an irreplaceable source for drug discovery [7]. Many conventional synthetic medicines are either directly derived from plant compounds or inspired by naturally occurring plant molecules. Well-known examples are artemisinin used for the treatment of malaria, silymarin for the treatment of liver diseases or cancer drugs such as paclitaxel and vincristine [7,8].

Medicinal and aromatic plants, especially those with ethnopharmacological practices, have also been used as a natural source for the treatment of diabetes. Traditionally, plant extracts and natural compounds have been shown to be effective in regulating hyperglycemia, thus helping to mitigate the progression of associated health complications. In addition to their therapeutic potential, these products are often relatively inexpensive and associated with minimal to no adverse side effects, making them an attractive alternative or adjunct to conventional treatments [9].

This review provides a comprehensive overview of the best-studied plants in the *Gentianaceae* family, highlighting their pharmacological importance and mechanisms of action in the treatment of diabetes and its associated complications. This review is not a systematic review; references were selected based on relevance and recentness through searches in PubMed and Scopus up to June 2025.

## 2. Plants from Gentianaceae Family: Traditional Use, Phytochemical Composition and Biological Activities

The plants from *Gentianaceae* family are divided into six tribes: *Chironieae*, *Exaceae*, *Gentianeae*, *Helieae*, *Potalieae* and *Saccifolieae*. The best-known genera of the *Gentianaceae* family include *Gentianella*, *Swertia*, *Centaurium*, *Blackstonia* and *Gentiana*, the largest and most widespread genus. The family includes flowering plants with 105 genera and about 1600 species, which occur worldwide and are mainly distributed in temperate and tropical regions [10].

### 2.1. Traditional Use of Gentianaceae

Medicinal herbs have been used for centuries as primary therapeutic tools, based on empirical knowledge in diverse cultures, and form the foundation of many traditional healing systems. The ethnomedicinal use of plants from the *Gentianaceae* family is widespread, in Chinese and Tibetan medicine [11], India [12], Ukraine [13], on the Balkan Peninsula [14,15], Peruvian medicine [16], and traditional Iranian medicine [17]. Plant extracts from the *Gentianaceae* family have been shown to have antibacterial, antioxidant, anticancer, anti-ischemic, antifibrotic and antiviral effects [17]. Traditionally, plants from the *Gentianaceae* family, both the roots and aerial parts, have been used to treat various health problems including gastrointestinal and liver disorders, skin diseases, wound healing, rheumatoid arthritis and diabetes [18]. The use of medicinal plants in Serbia for treating various health problems has a long history and is increasingly being studied [18]. Research on the use of two species, *Gentiana cruciata* and *Gentiana lutea*, among the population of Pirot County revealed that cross gentian (*Gentiana cruciata*) is highly popular and has diverse traditional uses [19]. These include treating diabetes, purifying the blood, cancer, boosting the immune system, increasing appetite, treating gastric and duodenal ulcers, high blood pressure, inflammation, lung diseases, colds, coughs, disease prevention, diseases of internal organs, high cholesterol, and leaking breast. The root of yellow gentian (*Gentiana lutea*) is used for improving diabetes, cancer, immune system, appetite, stomach health, blood purification, circulation, as an aphrodisiac, and against gastric and duodenal ulcers [19]. However, traditional use alone cannot consistently predict safety, highlighting the need for systematic evaluation of the pharmacological and toxicological properties of their active constituents. This is especially relevant for medicinal plants such as *Gentiana lutea*, whose bioactive monoterpenes may exhibit dual activities depending on concentration, cellular context, and interactions with other components of the prepared extract [20], which will be discussed in more detail later.

### 2.2. Phytochemical Composition and Biological Activity

The most important bioactive compounds in plants that contribute to the health benefits are secondary metabolites. These are natural low molecular weight organic compounds produced by plants that are not essential for growth and development, but are critical for survival, defense and adaptation to environmental stresses. Studies have shown that phenols (xanthones and C-glucoflavonoids) and terpenoids (secoiridoids) dominate the pharmacological background of the plants from the *Getianaceae* family [10,21,22,23,24]. The most abundant and best-studied secoiridoids are gentiopicroside and swertiamarin, which are found both in the aerial parts and in the roots of plants. The aerial parts also contain flavonoids, of which isoorientin and isovitexin are the most abundant [10]. The xanthones bellidifolin, isogentisin and mangiferin are most abundant in the roots [10]. The potential antidiabetic activity of *Gentianaceae* species is primarily attributed to their major constituents, such as gentiopicroside, swertiamarin and sweroside which have been shown to modulate glucose metabolism, oxidative stress and insulin signaling. In HFD/STZ-induced diabetic mice, gentiopicroside lowered blood glucose and improved insulin sensitivity by reducing hepatic gluconeogenesis and dyslipidemia, and also protected the liver and pancreas through anti-inflammatory and antioxidant activities [25]. Xiao and coworkers [26] reported that gentiopicroside reduced insulin resistance in HFD/palmitic acid-treated HepG2 cells by stimulating the PI3K/AKT signaling pathway. Swertiamarin also improves glycemic and lipid control and protects pancreatic beta cells from damage in STZ-diabetic rats [27]. The antidiabetic activity of swertiamarin, isolated from *Swertia pseudochinensis*, and its nitrogenous metabolites (R)-gentiandiol and (S)-gentiandiol, synthesized from swertiamarin, was investigated in type 2 KK/Upj-Ay mice fed a high-fat diet [28]. After a 7-day treatment with swertiamarin, (R)-gentiandiol and (S)-gentiandiol, the fasting blood glucose, total cholesterol, triacylglycerol, high-density lipoprotein cholesterol (HDL) and low-density lipoprotein cholesterol (LDL) were improved in the KKAy mice [28]. In the (R)-gentiandiol group, histological analysis of the kidney showed that the structure of the kidney tissue was completely restored and normal, while the structure of the kidney tissue in the (S)-gentiandiol group was very similar to that of the diabetic group [28]. Similar results were obtained for the pancreas. The structure of the pancreas was completely reestablished and normal in the (R)-gentiandiol group. These results suggest that (R)-gentiandiol exhibits a therapeutic effect and could be considered as a candidate for therapeutic use. Isolated secondary metabolite, sweroside from *Schenkia spicata* showed antidiabetic potential evaluated by measuring alpha-amylase and alpha-glucosidase inhibitory activities [29]. Xanthone fraction (1,7-dihydroxy-3,8-dimethoxyxanthone, 1,7-dihydroxy-3,4-dimethoxyxanthone, 1,7-dihydroxy-3,4,8-trimethoxyxanthone, 1,3,6,7-tetrahydroxyxanthene-9-β-D-glucopyranoside (mangiferin, MGF), and 1,3,6,7-tetrahydroxyxanthone (norathyriol, NTR)) isolated from the whole plants of *Gentiana acuta* effectively prevented obesity and hepatic steatosis in obese diabetic db/db mice (C57BLKS/J background Lepdb/Lepdb (db/db)) [30]. Histological analysis of epididymal adipose tissue showed reduced adipocyte area, while liver samples showed that hepatic steatosis was reduced in db/db mice [30]. In addition, hyperglycemia was reversed in db/db mice. It was found that NTR showed the best effects in lowering triglycerides and improving mitochondrial respiration among these xanthones. These authors showed that NTR modulated the activity and migration of dynamin-related protein 1 (Drp1) by regulating the orphan nuclear receptor subfamily 4 group A member, thereby attenuating the excessive mitochondrial fission induced by high glucose and fatty acid load. In addition, 7’-hydroxyl-substituted xanthones stimulated FUN14 domain containing 1 (FUNDC1)-mediated mitophagy, thereby promoting the clearance of dysfunctional mitochondria and maintaining mitochondrial homeostasis under hyperlipidemic conditions [30]. Various studies have shown the antidiabetic effects of other secondary metabolites contained in *Gentianaceae* species: flavonoids lower blood glucose levels, saponins, triterpenoids and steroid glycosides stimulate insulin secretion, and polysaccharides increase serum insulin levels and lower blood glucose levels [10,23,24,31].

## 3. Plant Extracts from Gentianaceae Family: Mechanistic Insights into Diabetes and Diabetic Complications

This section discusses the significant bioactive potential of plant extracts from the *Gentianaceae* family in in vivo (Table 1) and in vitro (Table 2) model systems, highlighting different mechanisms of action in the treatment of diabetes, including their antidiabetic effects, improvement of pancreatic beta cell function, antioxidant properties and modulation of lipid metabolism and comparison with standard antidiabetic drugs. Table 3 summarizes the effects of plant extracts from the *Gentianaceae* family on diabetic complications. 

### 3.1. Antidiabetic Effects

**Figure 2:**
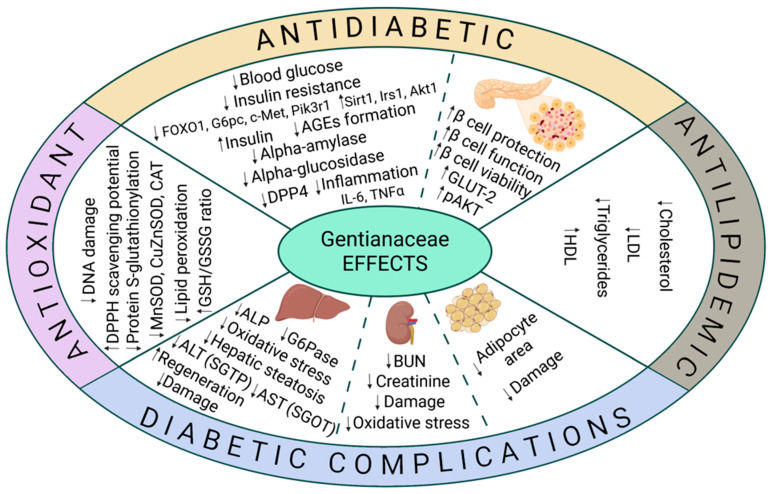
Biological effects of extracts and bioactive compounds derived from the *Gentianaceae* family. The schematic highlights their key targets and diverse mechanisms relevant to diabetes management, including antidiabetic effects, antioxidant properties, modulation of lipid metabolism, enhancement of pancreatic beta cell function, and documented protective actions against diabetes-related complications. AGEs—advanced glycation end products; ALT—alanine transaminase; AST —aspartate transaminase; BUN—blood urea nitrogen; SGOT—serum glutamate oxaloacetic transaminase; SGPT—serum glutamate pyruvic transaminase; ALP—alkaline phosphatase; GSH/GSSG—reduced to oxidized glutathione ratio; CAT—catalase; MnSOD and CuZnSOD—Mn- and CuZn- superoxide dismutase; G6pc—glucose-6-phosphatase catalytic subunit; c-Met—hepatocyte growth factor receptor; Pik3r1—phosphoinositide-3-kinase regulatory subunit 1; TNF-α—tumor necrosis factor-α; IL-6—interleukin-6; Akt1—alpha serine/threonine-protein kinase 1; Sirt1—Sirtuin 1; Irs1—insulin receptor substrate-1; Foxo1—forkhead box O1.

The extracts of *Gentiana dinarica* (aerial parts of wild plants, roots of wild plants, shoot cultures, vegetative root cultures, genetically transformed roots) and *Gentiana utriculosa* (aerial parts of wild plants, shoot cultures, genetically transformed shoots) demonstrated significant inhibition of intestinal alpha-glucosidase activity in vitro and exhibited antihyperglycemic activity in the oral glucose tolerance test in normoglycemic Wistar rats [32]. Potential therapeutic targets and mechanisms of *Gentianaceae* action for diabetes management are presented in Figure 2.

The other plant from the *Gentianaceae* family, *Centaurium erythraea*, is traditionally used in Morocco for the treatment of urinary retention, abdominal colic and diabetes mellitus [33,34]. Sefi and coworkers [34] showed a significant reduction in glucose levels and an increase in serum insulin levels in streptozotocin (STZ)-diabetic rats compared to diabetic rats in the study with oral administration of extracts from the leaves of *Centaurium erythraea*. Histological examination of the pancreatic tissue showed that treatment with *Centaurium erythraea* had a protective effect by increasing the area occupied by beta cells and the number and size of pancreatic islets [34]. The other study revealed that the oral administration of extracts from the aerial parts of *Centaurium erythraea* and *Artemisia herba*-alba to male C57BL/6J mice (fed a high-fat diet) lowered fasting blood glucose levels, plasma insulin concentrations and homeostasis model assessment (HOMA) levels [35]). Recent research with oral administration of *Centaurium erythraea* extract to diabetic rats (multiple low doses of STZ) improved insulin levels and glycemic control in diabetic rats [36]. The authors also showed an improvement of pancreatic tissue and a protective effect of *Centaurium erythraea* extract on the levels of insulin, glucose transporter 2 (GLUT-2) and phosphorylated protein kinase Akt in diabetic islets [36].

In addition to the hypoglycemic effects, the extract of *Centaurium erythraea* showed a positive effect in improving diabetic complications. The study with oral administration of *Centaurium erythraea* extract before (2 weeks) and/or after (4 weeks) induction of diabetes (i.e., pre-treatment and post-treatment, respectively) showed improved liver and kidney function in STZ-diabetic rats [37]. Both pre-treatment and post-treatment with *Centaurium erythraea* decreased the levels of liver enzymes (alanine transaminase (ALT) and aspartate transaminase (AST)) and markers of renal function (creatinine and blood urea nitrogen (BUN)) which were elevated in diabetic rats. In addition, the diabetic liver and kidney showed increased levels of O-linked N-acetylglucosamine (O-GlcNAc)-modified proteins, whereas post-treatment of the diabetic rats with *Centaurium erythraea* significantly reduced the levels of O-GlcNAc-modified proteins only in the liver. Pre-treatment with *Centaurium erythraea* reduced the O-GlcNAcylation of proteins in both the liver and kidney of diabetic rats [37].

In the STZ diabetes model in Swiss albino mice, the leaf extract of *Gentiana quadrifaria* showed an improvement in serum indicators of liver damage such as serum glutamate oxaloacetic transaminase (SGOT), serum glutamate pyruvic transaminase (SGPT) and alkaline phosphatase (ALP) [38]. In addition, a significant improvement in liver histology was observed, characterized by regenerative changes in hepatocytes, central vein and periportal areas compared to the untreated diabetic group [38].

The extract from the whole plant of *Swertia kouitchensis* inhibits alpha-amylase, alpha-glucosidase and insulin secretion in vitro [39]. Treatment of normal mice with this extract showed a remarkable inhibitory effect in the oral sucrose and starch tolerance test. This result suggested that the extract of *Swertia kouitchensis* could reduce postprandial glucose levels by inhibiting the activity of alpha-amylase and alpha-glucosidase, thus affecting the digestion of complex carbohydrates. Administration of *Swertia kouitchensis* to STZ-induced diabetic mice also increased serum insulin levels and lowered blood glucose levels (both acute and long-term effect) [39].

Previous studies have shown that the extract of *Swertia chirata* has anti-inflammatory, antipyretic, antiviral, hepatoprotective and anthelmintic effects [40,41]. The study with ethanolic extract of the leaf and its various fractions (pet-ether, dichloromethane and methanol fractions) of *Swertia chirata* in fasting Swiss albino mice showed that after 3 h of drug administration, the ethanolic extract of the leaf and the pet-ether fraction showed a remarkable blood glucose-lowering effect, while the dichloromethane and methanol fractions showed a mild to moderate blood glucose-lowering effect [42]. The other investigation with *Swertia chirata* root extract in alloxan-induced diabetic Wistar rats showed lowered blood glucose levels as well as serum levels of SGOT, SGPT and creatinine [43]. Giri and co-workers [44] selected eight medicinal plants—*Azadirachta indica*, *Gymnema sylvestre*, *Swertia chirata*, *Cinnamomum tamala*, *Psidium guajava*, *Aegle marmelos*, *Urtica dioica* and *Momordica charantia*—which have long been used for their glucose-lowering properties, and prepared four formulations to test the antihyperglycemic activity in vivo and in vitro. The obtained results showed that the formulation containing *chirata* exhibited potent in vitro inhibition of alpha-amylase and lowered blood glucose levels in alloxan-diabetic Swiss albino rats [44].

An extract from the aerial parts of *Gentiana olivieri* tested on STZ-induced diabetic Sprague-Dawley rats showed significant antihyperglycemic activity [37]. In addition, isolated flavonoid isoorientin from *Gentiana olivieri* showed a hypoglycemic effect on diabetic rats on the 15th and 25th day of subacute administration [45].

**Table 1:** The effects of *Gentaianceae* plant extracts in diabetes in vivo studies.

Species	Plant Part(s) Tested	Pharmacological Activity	Model/Diabetes Induction	Mechanism of Action	Standard Antidiabetic Drug/Comparison with Extract	Reference
*Gentiana dinarica*	aerial parts of wild growing plants/roots of wild growing plants/shoot culture/vegetative roots/genetically transformed roots	antidiabetic effects	Wistar rats	antihyperglycemic activity in the oral glucose tolerance test	no positive control	[32]
*Gentiana utriculosa*	aerial parts of wild growing plants/roots of wild growing plants/shoot culture/vegetative roots/genetically transformed roots	antidiabetic effects	Wistar rats	antihyperglycemic activity in the oral glucose tolerance test	no positive control	[32]
*Gentiana olivieri/* *Isoorientin*	aerial parts	antidiabetic effects	Sprague-Dawley rats/STZ	significant activity in glucose-hyperglycemic rats within 2 h after administration of extract/Isoorientin exhibited significant hypoglycemic and antihyperlipidemic effects	tolbutamide/similar effects of extract as tolbutamide	[45]
*Veratrilla baillonii*	aerial parts	antidiabetic, antioxidant and anti-inflammatory effects	Sprague-Dawley rats/high-sugar and high-fat diet with STZ	improvement of blood glucose and serum insulin levels/suppressed expression of many genes involved in insulin resistance/restored structure of pancreatic islet and the degree of dilatation of the lobular ducts and inflammatory infiltration	metformin/fasting blood glucose and serum insulin better than in metformin group/histology was similar to metformin group	[46]
*Veratrilla baillonii*	aerial parts	antidiabetic effects	Sprague-Dawley rats/high-fat diet	improvement of pathological damage of pancreas/insulin resistance: hypoglycemic effect by relieving insulin resistance	metformin/both extract and metformin significantly promoted weight gain, insulin resistance, ameliorated pathological damage of pancreas	[47]
*Swertia herbs (swertiamarin, (R)-gentiandiol and (S)-gentiandio)*	aerial parts/synthesized	antidiabetic and antihyperlipidemic effects	KKAy mice/High-Fat Diet	improvement of serum levels of total cholesterol, triglyceride, high-density and low-density lipoprotein cholesterol/improvement of histopathology of pancreas/identified 15 endogenous biomarkers, 10 of which were recalled by (R)-gentiandiol; between all biomarkers, glycine was the most effectively recalled by (R)-gentiandiol and regulated eight metabolic pathways	metformin/swertiamarin and (R)-gentiandiol showed similar level of protection as metformin	[28]
*Gentianella acuta*	whole plants/isolated xantones	antidiabetic effects	obese diabetic db/db mice	serum glucose, triglycerides, total cholesterol and free fatty acids were reduced	no positive control	[30]
*Gentianella bicolor*	whole plants	antidiabetic effects	Sprague-Dawley rat/STZ	decrease in blood glucose/pancreatic homogeneous structure with the presence of islets of virtually normal size	glibenclamide/blood glucose level better than glibenclamide	[16]
*Gentiana quadrifaria*	leaf	antidiabetic, antihyperlipidemic and antioxidant effects	Swiss albino mice/STZ	decrease in fasting blood glucose/reduction in serum low-density lipoprotein cholesterol, very-low-density lipoprotein cholesterol, total cholesterol and triglycerides and increase in high-density lipoprotein cholesterol levels/increased antioxidant enzymes catalase, superoxide dismutase, and glutathione reductase, and reduced serum glutamic-pyruvic transaminase, serum glutamic-oxaloacetic transaminase, alkaline phosphatase	ascorbic acid/metformin, glibenclamide, and insulin/similar level of protection of extract as positive controls	[38]
*Swertia kouitchensis*	whole plant	antidiabetic, antihyperlipidemic and antioxidant effects	BALB/c mice/STZ	decrease in blood glucose levels/increase in serum insulin levels/serum malondialdehyde, the activities of superoxidase dismutase and lipid levels (triglycerides, total cholesterol, low-density lipoprotein cholesterol, high-density lipoprotein cholesterol levels)	gliclazide/markers of carbohydrate metabolism and serum lipid profiles better than gliclazide/glucose level as gliclazide	[39]
*Swertia chirata*	root	antidiabetic effects	Wistar rats/alloxan	decrease in blood glucose levels	metformin/blood glucose level as in metformin	[43]
*Swertia chirata*	leaf and its pet-ether fraction	antidiabetic effects	Swiss albino mice	decrease in blood glucose level	glibenclamide/pet-ether fraction decreases blood glucose level similar to glibenclamide	[42]
*Swertia chirata*	aerial parts	antidiabetic effects	Swiss albino rats/alloxan	decrease in blood glucose level	glibenclamide/formulation that contained Swertia chirata showed blood glucose reduction similar to glibenclamide	[44]
*Enicostemma littorale*	whole plant	antidiabetic effects	rats/alloxan	decrease in blood glucose level	no positive control	[12]
*Enicostemma littorale*	whole plants	antidiabetic effects	Charles Foster rats/alloxan	improvement of blood glucose level, glycosylated hemoglobin, serum insulin	no positive control	[48]
*Enicostemma* *littorale*	whole plant	antidiabetic effects	Charles-Foster rats/alloxan/isolated rat pancreatic islets	enhance glucose-induced insulin release/increase in the serum insulin levels from isolated rat pancreatic islets/significant insulinotropic effect	glibenclamide/extract increased the serum insulin levels significantly at 8 h	[49]
*Enicostemma* *littorale*	whole plant	antidiabetic and antihyperlipidemic effects	human study/diabetic patients	reduced blood glucose and serum insulin levels/reduced urine sugar/serum cholesterol and serum triglycerides were significantly reduced and increase in serum high-density lipoprotein	no positive control	[50]
*Centaurium erythraea*	leaf	antidiabetic and antioxidant effects	Wistar rat/STZ	decrease in blood glucose/improvement of insulin, triglycerides and total cholesterol levels/pancreatic homogeneous structure with the presence of islets virtually normal size/improvement of oxidative stress parameters (malondialdehyde levels, protein carbonyl content, reduced glutathione content and enzymatic activities of superoxide dismutase, catalase and glutathione peroxidase in pancreas)	no positive control	[34]
*Centaurium erythraea*	aerial parts	antidiabetic and antioxidant effects	Wistar rat/STZ	improving the structural and functional properties of pancreatic islets/positive effects of extract on levels of insulin, glucagon, somatostatin, glucose transporter GLUT-2 and p-Akt	no positive control	[36]
*Centaurium erythraea*	aerial parts	antidiabetic and antioxidant effects	Wistar rat/STZ	improvement of serum alanine and aspartate aminotransferase, blood urea nitrogen and creatinine levels	no positive control	[37]
*Centaurium* *erythraea*	aerial parts	antidiabetic and antihyperlipidemic effects	C57BL/6J mice/high-fat diet	decrease in blood glucose/decreased insulin resistance/antihypercholesterolemic and antihypertriglyceridemic action	no positive control	[35]

*Veratrilla baillonii* is used for the intervention and treatment of a variety of diseases, including diabetes. In the study in which the application of *Veratrilla baillonii* extracts was conducted in Sprague-Dawley rats with high-fat diet/STZ model of T2D, the results showed that *Veratrilla baillonii* water extracts can improve liver damage and insulin resistance [47]. This treatment resulted in lowering blood glucose levels, improving glucose tolerance, reducing insulin resistance and improving pancreatic beta cell function. In addition, histological analyses showed the improvement of liver and pancreas after treatment with *Veratrilla baillonii* extract. These authors attempted to elucidate the antidiabetic mechanisms of *Veratrilla baillonii* extract and showed, by transcriptome analysis of the liver, that the treatment significantly suppressed the expression of many genes involved in insulin resistance and inhibited the levels of Foxo1, G6pc, c-Met and Pik3r1 in the liver while the expressions of genes related to metabolism and inflammation, including Sirt1, Irs1, Akt1, were significantly increased after treatment with *Veratrilla baillonii* extract. These findings suggest that *Veratrilla baillonii* extract holds considerable promise as a potential therapeutic candidate for diabetes treatment. The other study investigated the effect of *Veratrilla baillonii* extract in diabetic Sprague-Dawley rats (high-sugar and high-fat diet/low dose of STZ) [46]. After a 6-week treatment with *Veratrilla baillonii* extract, a significant hypoglycemic effect was observed by lowering fasting blood glucose levels and OGTT test, decreasing insulin resistance and improving pancreatic beta-cell function. In addition, histological analysis showed that treatment with *Veratrilla baillonii* extract reduced pathological damage in the liver, kidney, pancreas and epididymal adipose tissue by reducing inflammatory cytokines, such as IL-6 and TNF-α, as well as oxidative damage, fibrosis and inflammation [46].

The above-mentioned antidiabetic activity of swertiamarin and its nitrogenous metabolite (R)-gentiandiol revealed its protective effect on the kidneys and pancreas suggesting that (R)-gentiandiol could be considered as a candidate for therapeutic use [28]. The non-targeted serum metabolism method identified 15 endogenous biomarkers, 10 of which were recalled by (R)-gentiandiol. Between all biomarkers, glycine was the most effectively recalled by (R)-gentiandiol and regulated eight metabolic pathways. Those results indicate the promising potential of (R)-gentiandiol as an effective drug for the improvement of diabetes [28].

In traditional Peruvian medicine, *Gentianaceae* species, including *Gentianella bicolor*, have been used to treat diabetes since ancient times [16]. The evaluation of the hypoglycemic effect of the aqueous extract of *Gentianella bicolor* in diabetic Sprague-Downley rats treated with STZ showed a significant decrease in blood glucose levels after 21 days of treatment compared to the diabetic group. In addition, histopathological examination showed a regenerative effect of the pancreatic islets after treatment with the aqueous extract of *Gentianella bicolor*, as the pancreas of this experimental group showed a homogeneous structure with islets of practically normal size [16].

*Enicostemma littorale* is used as an antidiabetic agent by the rural population of Gujarat and other parts of India [12]. The aqueous whole plant extract of *Enicostemma littorale* showed a significant decrease in blood glucose levels associated with a decrease in glycosylated hemoglobin and glucose-6-phosphatase activity in the liver after a 30-day treatment of alloxan-induced diabetic rats [12]. The other study with the extract of whole *Enicostemma littorale* was tested on alloxan-induced diabetic rats (Charles Foster rats) and the results showed that *Enicostemma littorale* caused a significant reduction in blood glucose levels and an increase in serum insulin levels [48]. Maroo and coworkers [43] showed that single administration of an aqueous extract of *Enicostemma littorale* to alloxan-induced diabetic rats (Charles Foster rats) increased serum insulin levels significantly and comparably to the glibenclamide-treated diabetic group. Furthermore, the aqueous extract of *Enicostemma littorale* has the potential to increase glucose-induced insulin release in isolated rat pancreatic islets, probably via an ATP-sensitive potassium channel dependent pathway independent from Ca^2+^ influx [49]. *Enicostemma littorale* is also a widely used Ayurvedic remedy for the treatment of diabetes. In a study by Alqarni and coworkers [51], the alpha-amylase inhibitory activity of *Enicostemma littorale* was demonstrated. Moreover, *Enicostemma littorale* showed an inhibitory effect on dipeptidyl peptidase-4 (DPP-IV) in an in vitro enzyme assay [45]. Since glucagon-like peptide 1 (GLP-1)-based therapies control glucose levels via DPP-4, these results indicate the potential of *Enicostemma littorale* as a source for the further development of new antidiabetic agents [52]. 

### 3.2. Antioxidant Properties

Extracts of *Gentiana dinarica* (aerial parts of wild plants, roots of wild plants, shoot cultures, vegetative root cultures, genetically modified roots) and *Gentiana utriculosa* (aerial parts of wild plants, shoot cultures, genetically modified shoots) showed the ability to scavenge DPPH free radicals in vitro [32]. Using the comparative HPLC method, before and after the reaction with DPPH radicals, compounds responsible for free radical scavenging activity were identified. In the *Gentiana dinarica* extract, xanthones, norswertianin-1-O-primeveroside and norswertianin, while in *Gentiana utriculosa* xanthone, mangiferin, decussatin, and decussatin-1-O-primeveroside had the highest ability to scavenge free radicals [32].

The extract of *Centaurium erythraea* was analyzed in vitro in insulinoma Rin-5F beta cells treated with STZ. The results showed that oxidative stress leads to disturbances after STZ treatment and that the application of *Centaurium erythraea* extract leads to a reduction in DNA damage, lipid peroxidation, S-glutathionylation of proteins and a reduction in antioxidant enzyme (MnSOD, CuZnSOD and CAT) activities [36]. In addition, treatment with *Centaurium erythraea* extract improved the transcriptional regulation of antioxidant enzymes (CAT, MnSOD, CuZnSOD, GPx and GR) in beta cells, as well as adjusting the presence and activities of redox-sensitive transcription factors (NFκB-p65, FOXO3A, Sp1 and Nrf-2) [36]. The other study investigated the effect of *Centaurium erythraea* extract against H_2_O_2_- and SNP-induced oxidative/nitrosative stress in Rin-5F beta cells [53]. The results showed the antioxidant activity of *Centaurium erythraea* extract by reducing DNA damage, lipid peroxidation and protein S-glutathionylation, restoring ratio of reduced to oxidized glutathione (GSH/GSSG ratio) and antioxidant enzymes (CAT, GPx, GR, MnSOD and CuZnSOD) activities, mRNA and protein levels [46]. It was also shown that *Centaurium erythraea* extract increased insulin expression/secretion, especially in H_2_O_2_-treated beta-cells. These results were in line with the more prominent antioxidant effect of *Centaurium erythraea* in H_2_O_2_-treated beta-cells compared to SNP-treated cells [53]. Beside antihyperglicemic and antioxidant properties, use of *Centaurium erythraea* extract revealed improvement in diabetic complications, such as liver and kidney functioning. Lower levels of lipid peroxidation, DNA damage and protein glutathionylation, an increased GSH/GSSG ratio and attenuated disruption of antioxidant enzyme activities indicated the antioxidant effect of *Centaurium erythraea* extract in the diabetic liver and kidney [53]. In an in vivo study with oral administration of *Centaurium erythraea* leaf extracts to STZ-diabetic rats, the results showed decreased lipid peroxidation and protein oxidation as well as increased GSH content in pancreatic tissue compared to the diabetic rats [34]. In addition, the decreased activity of antioxidant enzymes (SOD, CAT and GPx) was restored in the diabetic rats and showed a significant increase in the activities of these enzymes in the pancreatic tissue after the administration of *Centaurium erythraea* [34].

**Table 2:** The effects of *Gentaianceae* plant extracts in diabetes in vitro studies.

Species	Plant Part(s) Tested	Pharmacological Activity	Model	Mechanism of Action	Reference
*Gentiana dinarica*	aerial parts of wild growing plants/roots of wild growing plants/shoot culture/vegetative roots/genetically transformed roots	antioxidant/antidiabetic effects	in vitro testing of extract	scavenge DPPH free radicals/inhibition of intestinal α-glucosidase in vitro/norswertianin-1-O-primeveroside and norswertianin have the highest scavenging potential	[32]
*Gentiana utriculosa*	aerial parts of wild growing plants/roots of wild growing plants/shoot culture/vegetative roots/genetically transformed roots	antioxidant/antidiabetic effects	in vitro testing of extract	scavenge DPPH free radicals/inhibition of intestinal alpha-glucosidase in vitro/mangiferin, decussatin, and decussatin-1-O-primeveroside the highest scavenging potential	[32]
*Centaurium erythraea*	aerial parts	antioxidant/antidiabetic effects	Rin-5F b-cells/STZ	reduced DNA damage, lipid peroxidation, protein S-glutathionylation/improvement of enzyme activity and transcriptional regulation of catalase, Mn superoxide dismutase, CuZn superoxide dismutase, glutathione peroxidase and glutathione reductase enzyme/promoting proliferative and pro-survival pathways in beta-cells: readjustment of the presence and activities of redox-sensitive NFκB-p65, FOXO3A, Sp1 and Nrf-2; fine-tuned modulation of the activities ofAkt, ERK and p38 kinases and of Pdx-1 and MafA regulatory factors	[36]
*Centaurium erythraea*	aerial parts	antioxidant effects	Rin-5F b-cells/H2O2- and SNP-induced oxidative/nitrosative stress	increase cell viability and ameliorate the disturbance of redox homeostasis in H2O2- and SNP-treated cells by decreasing DNA damage, lipid peroxidation and protein S-glutathionylation/adjustment of mRNA, protein levels and activities of glutathione peroxidase, glutathione reductase, Mn superoxide dismutase, CuZn superoxide dismutase and catalase/preventing or slowing down beta-cell damage and dysfunction caused by oxidative/nitrosative stress	[53]
*Enicostemma littorale*	aerial parts	antioxidant/antidiabetic effects	in vitro testing of extract	DPPH radical scavenging activity/hydrogen peroxide scavenging activity/promising activity based on in vitro dipeptidyl peptidase-IV inhibitors	[52]
*Enicostemma littorale*	aerial parts	antidiabetic effects	in vitro testing of extract	alpha-amylase inhibition/in vitro drug release: transdermal delivery of capsulated extract on rat skin	[51]
*Schenkia spicata*	isolated sweroside	antioxidant/antidiabetic effects	in vitro testing of extract	in vitro alpha-amylase and alpha-glucosidase inhibitory activities/in vitro free radical scavenging and antioxidant potential (ABTS, CUPric Reducing Antioxidant Capacity and Ferric Reducing Antioxidant Power assays)	[29]
*Gentiana quadrifaria*	leaf	antioxidant effects	in vitro testing of extract	free radical scavenging capability: DPPH and ABTS assays/total phenolic content/total flavonoid content	[38]
*Swertia kouitchensis*	whole plant	antidiabetic effects	in vitro testing of extract/NIT-1cell	in vitro α-amylase and α-glucosidase activity test/In vitro NIT-1 cell insulin secretion test	[39]
*Swertia chirata*	aerial parts	antioxidant/antidiabetic effects	in vitro testing of extract	DPPH free-radical scavenging activity/in vitro inhibition of alpha-amylase	[44]

In vitro studies have shown that the extract of *Enicostemma littorale* scavenges DPPH radicals and H_2_O_2_. The extract of *Enicostemma littorale* was tested on alloxan-induced diabetic rats (Charles Foster rats) and the results showed an antioxidant effect by improving blood GSH levels, CAT activity in erythrocytes and lipid peroxidation [48].

In the study in which the isolated swerosides of *Schenkia spicata* were evaluated by various in vitro assays (ABTS, CUPric Reducing Antioxidant Capacity (CUPRAC) and Ferric Reducing Antioxidant Power assay (FRAP)), a high free radical scavenging and antioxidant potential was confirmed [29].

The extract from the leaves of *Gentiana quadrifaria* showed high free radical scavenging potential under in vitro conditions (DPPH and ABTS assays) which was attributed to the high content of phenols and flavonoids [38]. In in vivo experiments, the leaf extract of *Gentiana quadrifaria* increased the activity of antioxidant enzymes (CuZnSOD and MnSOD, catalase and GR) and decreased the protein carbonyl content in the liver of diabetic Swiss albino mice [38].

Administration of *Swertia kouitchensis* to STZ-induced diabetic mice resulted in the reduction in serum malondialdehyde and improvement of serum SOD activity [39].

Giri and coworkers [44] revealed potent DPPH free-radical scavenging effect in the formulations with eight selected medicinal plants—*Azadirachta indica*, *Gymnema Sylvestre*, *Cinnamomum tamala*, *Psidium guajava*, *Aegle marmelos*, *Urtica dioica*, *Momordica charantia* and one member of *Gentianaceae* family, *Swertia chiraita*.

**Table 3:** The effects of *Gentaianceae* plant extracts in diabetic complications.

Species	Plant Part(s) Tested	Model/Diabetes induction	Targeted Organ	Mechanism of Action	Standard Antidiabetic Drug/Comparation with extract	Reference
*Veratrilla baillonii*	aerial parts	Sprague Dawley/high-sugar and high-fat diet with STZ	liver, kidney, epididymal adipose tissue	inhibited the levels of Foxo1, G6pc, c-Met and Pik3r1 in the liver while the expressions of genes related to metabolism and inflammation, including Sirt1, Irs1, Akt1, were significantly increased/restored structure of pancreatic islet and the degree of dilatation of the lobular ducts and inflammatory infiltration were also alleviated/epididymal adipose tissue showed that the size of adipocytes decreased and the density of adipocytes increased/improvement of kidney histology and fibrosis/α-SMA expression in the liver was significantly reduced and improved liver fibrosis/superoxide dismutase and glutathione peroxidase were increased, malondialdehyde was lowered in the liver/decrease in the indicators of inflammation, IL-6 and TNF-α, in the liver/improved glucose metabolism through modulating the IRS1/PI3K/AKT pathway	metformin/histology and signaling pathways were similar to metformin group	[46]
*Veratrilla baillonii*	aerial parts	Sprague-Dawley rats/high-fat diet	liver	improvement of pathological damage on liver	metformin/both extract and metformin significantly ameliorated pathological damage of liver	[47]
*Swertia herbs (swertiamarin, (R)-gentiandiol, and (S)-gentiandiol)*	aerial parts/synthesized	KKAy mice/High-Fat Diet	kidney	improvement of histopathology of kidney	metformin/swertiamarin and (R)-gentiandiol showed similar level of protection as metformin	[28]
*Gentianella acuta*	whole plants/isolated xantones	obese diabetic db/db mice	liver, epididymal adipose tissue	size and weight of adipose deposits in epididymal adipose tissue/hepatic steatosis was reduced/GeneChip profiling of the liver transcriptome revealed potential activity on stimulating autophagy and regulating mitochondrial function/activity and migration of dynamin-related protein 1 (Drp1) by regulating the orphan nuclear receptor subfamily 4 group A member, thereby attenuating the excessive mitochondrial fission induced by high glucose and fatty acid load	no positive control	[30]
*Gentiana quadrifaria*	leaf	Swiss albino mice/STZ	liver	increased antioxidant enzymes catalase, superoxide dismutase, and glutathione reductase, and reduced serum glutamic-pyruvic transaminase, serum glutamic-oxaloacetic transaminase, alkaline phosphatase and protein carbonyl in liver/improvement of liver histology	ascorbic acid/metformin, glibenclamide and insulin/similar level of protection of extract as positive controls	[38]
*Swertia chirata*	root	Wistar rats/alloxan	liver, kidney	decrease in blood glucose levels/ameliorated the pathological condition of liver and kidney (liver and kidney functioning tests)	metformin/liver and kidney functioning tests similar to the metformin group	[43]
*Enicostemma littorale*	whole plant	rats/alloxan	liver	decrease in the level of glycosylated hemoglobin and glucose-6-phosphatase activity in liver	no positive control	[12]
*Enicostemma littorale*	whole plants	Charles Foster rats/alloxan	liver	improvement of liver glucose 6-phosphatase, catalase, lipid peroxidation and reduced glutathione	no positive control	[48]
*Enicostemma littorale*	whole plant	human study/diabetic patients	kidney, cardiovascular system	improvement of kidney function, lipid profile and blood pressure	no positive control	[50]
*Centaurium erythraea*	aerial parts	Wistar rat/STZ	liver, kidney	reduced DNA damage in the liver and kidney estimated by Comet assay/improvement of liver and kidney catalase, superoxide dismutase, glutathione peroxidase, glutathione reductase enzyme activities and protein levels/decreased level of posttranslational O-GlcNAc modified protein in liver and kidney	no positive control	[37]

### 3.3. Improvement of the Lipid Profile

In the study in which an extract from the leaves of *Gentiana quadrifaria* was administered to STZ-diabetic Swiss albino mice, the obtained results showed a significant reduction in total cholesterol, LDL-cholesterol (LDL-C), very-low-density lipoprotein cholesterol (VLDL-C) and triglycerides associated with an increase in HDL-cholesterol (HDL-C) in diabetic mice [38].

Plasma triglyceride and total cholesterol concentrations were significantly lower at the end of the study after oral administration of an extract of the aerial parts of *Centaurium erythraea* and *Artemisia herba* to male C57BL/6J mice fed a high-fat diet, while no significant difference was observed in plasma HDL-C concentrations between the controls and treated groups [35]. The other study with oral administration of *Centaurium erythraea* leaf extracts to STZ-diabetic rats significantly reduced total cholesterol and triglyceride levels, which were elevated in the diabetic group [34].

After treatment of STZ-induced diabetic mice with *Swertia kouitchensis* extract, triglycerides, total cholesterol and LDL levels were decreased while HDL levels were increased [39].

The antihyperlipidemic effect of isoorientin, isolated from the aerial parts of *Gentiana olivieri*, was demonstrated in STZ-induced diabetic rats on the 15th and 25th day of subacute administration [45].

The xanthone fraction (1,7-dihydroxy-3,8-dimethoxyxanthone, 1,7-dihydroxy-3,4-dimethoxyxanthone, 1,7-dihydroxy-3,4,8-trimethoxyxanthone, 1,3,6,7-tetrahydroxyxanthene-9-β-D-glucopyranoside (mangiferin, and 1,3,6,7-tetrahydroxyxanthone (norathyriol, NTR)) isolated from the whole plants of *Gentiana acuta* were able to reverse hyperlipidemia in db/db mice [30].

The antidiabetic activity of swertiamarin, isolated from *Swertia pseudochinensis,* and its nitrogenous metabolites (R)-gentiandiol and (S)-gentiandiol, synthesized from swertiamarin, were investigated in KK/Upj-Ay type 2 mice fed a high-fat diet [41]. After a 7-day treatment, the total cholesterol, triacylglycerol, HDL-C and LDL-C of KKAy mice were improved, while (R)-gentiandiol showed a noticeable improvement, implying being a potential candidate for further investigations as a therapeutic drug [28].

## 4. Bioavailability Challenges and Toxicity of the Most Abundant Phytoconstituents of Gentianaceae Family

Providing the intelligent dissolution and consistent delivery of bioactive compounds is a current challenge receiving considerable attention to align with desired therapeutic goals [54]. Although the main constituents of the *Gentianaceae* family, such as gentiopicroside, swertiamarin and sweroside exhibit a wide range of biological activities, their absorption, distribution and biotransformation (in humans) may be inefficient due to limited bioavailability and metabolism which can also produce metabolites with toxic effects. Moreover, the inconsistency between the activity of isolated secoiridoids and crude extracts cannot be ignored. Therefore, when evaluating potential toxicity, both the bioavailability of these compounds and the composition of the extracts must be considered, as these factors influence their biological effects and may either increase or reduce the toxic effects observed in vitro [55]. The main advantages of encapsulating bioactive compounds in various carriers are to improve their stability, protection from gastrointestinal environment and controlled and extended release [56]. For example, the health benefits of gentiopicroside could be enhanced by using new formulations such as liposomes, microencapsulation or nanoparticles to increase its bioavailability and distribution to target tissues [57]. More importantly, new formulations of gentiopicroside may enable its evaluation in clinical trials, providing the basis for studies on bioactivity, efficacy, and potential toxicity or safety in metabolic disorders [55]. Sobot and colleagues [20] conducted a toxicological evaluation of *Gentiana lutea* L. root extract (GE) and its monoterpene compounds because gentiopicroside exhibits dual effects, ranging from cytoprotection to cytotoxicity depending on experimental conditions, while the roles of sweroside and swertiamarin range from non-toxic to markedly cytotoxic. In their study, in silico predictions were combined with in vitro cyto- and genotoxicity assays on human primary peripheral blood mononuclear cells (PBMCs) to assess the toxic potential of these monoterpenes. These compounds significantly reduced cell viability and increased DNA damage, while GE induced genotoxicity only in unstimulated PBMCs, not in mitogen-stimulated cells. The milder effects of GE, despite its higher gentiopicroside content, suggest that the complexity of the whole extract may mitigate the toxicity of individual compounds. Such evidence indicates that herbal products, although generally considered safe due to their long history of use, have complex phytochemical profiles that can produce both beneficial and adverse biological effects, underscoring the need for careful and systematic toxicological evaluation.

### Clinical Evidence on the Therapeutic Application of Plant Extracts from Gentianaceae Family

The previously discussed results from in vitro experiments and animal models indicate promising potential for plant extracts from the *Gentianaceae* family in treating diabetes and its complications. However, clinical research on the therapeutic applications of *Gentianaceae* plant extracts in diabetic patients is very limited. The efficacy of *Enicostemma littorale* was investigated in patients with T2D (twice-daily tablets over three months) [50]. The results of this clinical trial showed reductions in blood glucose, serum insulin, and urine glucose levels. The treatment also led to significant reductions in pulse rate, systolic and diastolic blood pressure, and serum creatinine levels. *Enicostemma littorale* improved the lipid profile: after three months of treatment, serum cholesterol and serum triglycerides were significantly reduced, while serum HDL was significantly increased [50]. This clinical study is promising and provides a basis for future research that should focus on well-designed trials to examine and confirm the safety and efficacy of *Gentianaceae* in human populations.

However, the reported adverse effects associated with *Gentiana lutea* highlight the need for cautious and systematic toxicological assessment in future clinical investigations. Despite their promising pharmacological potential, the therapeutic use of *Gentiana* species remains limited by insufficient clinical evidence, uncertainties about safety at higher doses, and the possibility of drug–herb interactions, especially with medications metabolized through hepatic or glucose-regulatory pathways. (e.g., gentiopicroside and glucose control) [21]. 

## 5. Current Limitations, Research Gaps and Future Perspectives

Extracts from plants belonging to the *Gentianaceae* family have received increasing attention as potential natural agents for the treatment of diabetes and its associated complications. A large number of in vitro and in vivo studies have shown that they are able to regulate blood glucose levels, reduce oxidative stress, protect against tissue and organ damage and improve lipid metabolism. In addition, several phytochemicals with antidiabetic activity have been successfully isolated and characterized from *Gentianaceae* species, further highlighting the pharmacological importance of this plant family. Their long-standing use in traditional medicine in various cultures provides further justification for their continued exploration as complementary or alternative therapies. Compared to synthetic medicines, herbal preparations are often considered more accessible, less expensive and associated with fewer adverse effects. The frequent use of whole plant extracts in experimental studies is particularly noteworthy, as the synergistic interaction of multiple ingredients likely enhances their therapeutic efficacy beyond that of single isolated substances. Despite these promising results, the current evidence still has significant limitations. Most available studies have been conducted in vitro or in animal models, while robust clinical studies are still deficient. This gap significantly limits the translation of preclinical results into clinical practice. Furthermore, the mechanisms underlying the observed effects are only partially understood. Another challenge lies in the variability of phytochemical composition depending on species, growing conditions and extraction methods, which makes reproducibility and standardization difficult.

Apart from pharmacological considerations, the conservation of species from the gentian family poses a critical challenge. This family is a reservoir of chemical diversity that contains numerous known bioactive compounds and offers enormous potential for the discovery of new therapeutic agents. However, global biodiversity assessments show that climate change and habitat loss pose the greatest threats to plant survival. Shifts in distribution patterns due to changing climatic conditions and habitat destruction from agricultural expansion and invasive species are accelerating the loss of biodiversity at an alarming rate. Without effective conservation and sustainable use strategies, the uncontrolled exploitation of *Gentianaceae* family species could lead to irreversible decimation or extinction. This would not only endanger ecological stability, but also preclude valuable opportunities for drug discovery and development.

In conclusion, the *Gentianaceae* family has considerable potential for the development of new therapeutic strategies against diabetes and its complications. To fully exploit this potential, future research should focus on well-designed clinical trials to confirm safety and efficacy in human populations, studies at the molecular and cellular level, standardization of extraction and characterization protocols to ensure reproducibility, and integration of principles of conservation biology into pharmacognostic research. If these challenges are overcome, plants from the *Gentianaceae* family can move from traditional remedies and experimental findings to validated, sustainable and clinically applicable interventions for diabetes treatment.

## Data Availability

No new data were created or analyzed in this study.
